# The Efficacy of Self-care Behaviors, Educational Interventions, and Follow-up Strategies on Hospital Readmission and Mortality Rates in Patients with Heart Failure

**DOI:** 10.31661/gmj.v12i.3116

**Published:** 2023-12-01

**Authors:** Zahra Khosravirad, Mohammad Rostamzadeh, Shiva Azizi, Mehran Khodashenas, Babak Khodadoustan Shahraki, Farangis Ghasemi, Maryam ghorbanzadeh

**Affiliations:** ^1^ Department of Quality Improvement in Kowsar Hospital, Shiraz, Iran; ^2^ Department of Cardiology, School of Medicine, Ardabil University of Medical Sciences, Ardabil, Iran; ^3^ Department of Nursing, School of Nursing, North Khorasan University of Medical Sciences, Bojnurd, Iran; ^4^ School of Medicine, Alborz University of Medical Sciences, Karaj, Iran; ^5^ Isfahan University of Medical Sciences, Isfahan, Iran; ^6^ Department of Biology, Jahrom Branch, Islamic Azad University, Jahrom, Iran

**Keywords:** Self-care, Heart Failure, Education, Patient Readmission, Hospitalization

## Abstract

Heart failure (HF), a worldwide epidemic with significant morbidity and mortality risks, is frequently secondary to cardiovascular disorders and probably is the common final way to survive patients. Almost 25% of hospitalized patients with acute HF are expected to be readmitted within 30 days post-discharge, and the rates of rehospitalization increase to almost one-third at 60 days and 60 percent within one year of discharge. Although care planning for patients with heart failure is complex, multidisciplinary, and resource-dependent, optimal self-care management along with appropriate educational intervention and follow-up strategy could be able to reduce readmissions, decline the duration of hospitalization, increase life expectancy, decrease the rates of mortality, and reduce costs of healthcare services for patients with HF. However, there are contradictions in previous reports about the efficacy of self-care, mainly due to patients’ non-adherence to self-care behaviors. Therefore, the current study aimed to review the investigations on the effectiveness of self-care of HF patients in reducing hospital readmissions and increasing quality of life, and discuss novel approaches for predischarge educational interventions and postdischarge follow-up strategies.

## Introduction

Heart failure (HF) is considered a worldwide epidemic with significant morbidity and mortality risks [1]. It is widely evidenced that HF manifests complicated clinical syndromes characterized by both typical symptoms, including shortness of breath, tachypnea, fatigue, and ankle swelling as well as additional symptoms including pulmonary crackles, elevated jugular venous pressure, and peripheral edema all of which result from an abnormal cardiac structure and function [2][3]. These abnormalities lead to a significant reduction in cardiac output and increased intracardiac pressures during stress or at rest [2][3]. It is estimated that more than 30 million people suffer from HF all over the world. It is important to note that, the annual risk of HF increases tenfold between the ages of 60 and 90 and the risk of death is approximately 50% within five years of diagnosis [4][5]. Although it was believed that the incidence of HF has decreased in recent years, the comparison of epidemiological studies over the past two decades suggests the opposite of this belief [5][6], and the real burden, specifically in non-western countries, is under-reported in mortality and self-report data [7].

It is evidenced that HF is frequently secondary to cardiovascular disorders (CVDs), particularly coronary artery disease (CAD), and probably is the end stage of cardiac complications [8]. The progression of HF is assumed to be unpredictable, however, it is mainly characterized by a downward trajectory of a functional drop with constant periods marked by acute decompensation [9]. As mentioned earlier, HF is considered an age-related disease, hence patients with HF represent a significant burden of comorbid diseases, including type 2 diabetes and renal failure, and cognitive decline, which both are considered leading causes of hospital readmissions as well as the concomitant trajectory for CVD and HF. Therefore, care planning and delivery in HF is considered complex, multidisciplinary, and resource-dependent.

t is demonstrated that unplanned readmissions of 30-day-all-cause are significantly high in the setting of HF. For example, admitting approximately one million HF patients in the United States resulted in a cost of more than US$30 [10]. Notably, almost 25% of patients with acute HF admitted to the hospital are expected to be readmitted within 30 days post-discharge, and the majority of them are readmitted within the first two weeks. In addition, the rates of readmission increase to nearly one-third at 60 days and 60 percent within one year of discharge [11][12]. Interestingly, in patients with HF, readmissions less than 90 days have been attributed to CVD factors, while readmissions longer than this time frame are more often attributed to comorbidities [13][14]. Although the reports presented in recent years indicated a significant decrease in the rates of hospital readmission more than 30 days after discharge, in-hospital mortality, and the length of stay for patients with HF, the reduction in 30-day readmissions was not significant. [15][16]. Inconclusive evidence has led researchers to assume different factors including inherent problems with the clinical indicator, inadequate progress with improving discharge planning, and insufficient improvement in transitional care contribute to this issue. It is documented that 50 percent of readmissions of patients with HF are preventable and most of them are attributed to poor adherence to the HF management program [15][16], thereby the pivotal contribution of patient self-care in secondary management appears.

According to what was aforementioned, proper self-care management is necessary to reduce readmissions, decline the duration of hospitalization, increase the life expectancy of patients with HF, and reduce healthcare costs. However, to more precisely assess this efficacy, the present study aimed to review the merits and challenges of self-care management in patients with HF.

Self-care of Patients with HF

Intensive and dynamic resources are one of the most important needs of complicated self-care of patients with HF, which are vitally required to be updated and regular and need continuous self-care programs, as well as a strong physician-patient alliance [17][18]. Currently, several HF management guidelines recommend a seamlessly coordinated and fully integrated system of care across multidisciplinary healthcare services for the disease, all tailored to the individual needs of patients and their local context [19]. Accordingly, these guidelines emphasize the clinical significance of patient self-care and describe it as the process of maintaining the health of patients with HF through health-promoting measures, reducing the risk of re-hospitalization, and disease management through maintenance, monitoring, and behavioral care [20][21].

Recently, the Association of the European Society of Cardiology declared that self-care is an undeniable necessity in the long-term management of chronic HF and described the relevant guidelines as emphasizing the importance of patient education about adherence to medication, changes of lifestyle, monitoring of symptoms, and sufficient response to the potential deterioration [22]. Experts in the field of self-care of HF patients have assumed that self-care is associated with medical and person-centered outcomes in patients with HF, including improved quality of life, as well as lower rates of re-hospitalization and mortality. Therefore, experts believe that self-care of patients with HF emphasizes behaviors that are related to adherence, including medication prescriptions and maintaining lifestyle, and risk assessment, including recognition, evaluation, management, and appropriate action on particular symptoms of HF [23][24]. Nevertheless, it is clear that the published guidelines provide general guidance for self-care recommendations, hence healthcare professionals working with patients with HF require more specific recommendations [22]. Consequently, guidelines on physical activity, immunization and infection prevention, medication adherence, mental status, nutrition, sleep, leisure and travel, smoking, symptom management, and symptom monitoring, as well as the efficacy of each subject appear to be necessary.

The Efficiency of Self-care Interventions on Clinical Outcomes of HF Patients

The possibility of educating HF patients to note and monitor specific symptoms at home for 30 days has been revealed and indicated that self-care behavior significantly improved after 30 days of using symptom notes at home [25]. Healthcare providers involved in the management of HF widely benefit from interventions designed to equip patients with the knowledge and skills needed to monitor and manage their condition and optimize modifiable risk factors [26]. The conducted randomized control trial widely suggested that self-care interventions can potentially improve the clinical outcomes of HF patients including significantly reducing the rate of hospital readmissions. For instance, it is demonstrated that self-care education in patients with HF remarkably decreases the risk of unexpected re-hospitalization at twelve months by 30% [27].

Contradictory, several recent studies have found that self-care interventions did not improve the rate of hospital readmissions in patients with HF. In this regard, a randomized controlled trial evaluating the efficacy of the self-care intervention on 90-day outcomes in 479 patients with acute HF and a median age of 63.0 years revealed that self-care interventions could not improve the primary global rank outcome during 90 days [28]. However, desired outcomes during 30 days may suggest that an early benefit of tailored self-care management is not sustained through 90 days [28]. However,a randomized controlled trial on 196 congestive HF patients to assess 30-day readmission demonstrated that reduced readmission rates were not significantly correlated with better-reported patient self-care behavior, heart failure knowledge, and medication compliance [29].

Therefore, there are documented inconsistencies in the reports regarding the effectiveness of self-care in reducing the rate of hospital readmission of patients with HF. Professionals believe that poor patient adherence to self-care interventions mainly explains some of these inconsistencies. In this regard, systematic reviews and meta-analyses have helped to understand the contradictions by showing that self-care interventions emphasizing medication adherence can lead to a significant reduction in mortality risk and reduce hospital readmission rates in patients with HF [30][31]. Although these studies have documented the benefit of self-care interventions on HF-related readmissions, less consistency in reducing mortality risk has been reported [30][31]. Interestingly, in recent years, various studies have been conducted to introduce novel self-care methods and determine their effectiveness, which are discussed below.

Effectiveness of HF Patient Self-care Education and Follow-Up

As discussed earlier, the established clinical guidelines for HF from all over the world have recommended that self-case is a pivotal factor in avoiding preventable hospital readmission rates and achieving optimal outcomes for patients with HF [32][33]. In this regard, knowledge is considered a crucial factor influencing the behaviors of HF patients’ self-care. It is demonstrated that limited knowledge of health care services for patients with HF may be associated with both inappropriate self-care and restricted service application highlighting the significance of educating patients with HF [34]. It has consistently been traditional to provide patients with printed information about disease characteristics, including symptoms, risks, and related self-care for bedside education. Nevertheless, the printed format is not always recommended because many mismatches have been documented in this case, which was generally due to mistakes in printed information, illiteracy of the patients, or insufficient health knowledge of the patient [35][36]. Indeed, the lack of optimal knowledge and appropriate self-care contributes to poor outcomes and hospital readmission for people with HF. Therefore, evaluating the efficacy of self-care education, educational methods, and follow-up of patients with HF has been a concern of researchers in recent years.

According to what was aforementioned, various studies have evaluated the effectiveness of different educational methods in improving self-care behavior looking for reducing the readmission of HF patients. A recent pragmatic randomized controlled trial on 36 HF patients with an age of 67.5±11.3 years old revealed that at 90 days, the intervention group participants, who used the avatar education application to learn self-care behavior had a higher increase in knowledge score [35][36].

Since it is believed that modifications of lifestyle behavior may promote the self-management of chronic cardiac diseases and improve quality of life, researchers were keen to find whether a mobile health program can assist patients with HF in the self-management of the disease during the acute posthospital discharge period. In this regard, a randomized controlled trial on 31 HF patients with an average age of 60.4 demonstrated that an educational mobile health program tailored for patients with HF is feasibly deployed and acceptable by patients resulting in better HF prognosis by a higher mean quality of life at 30 days posthospitalization and a longer duration before hospital readmission [37]. In addition to mobile health programs, a pilot study showed that computer-based intervention can improve self-care in patients with HF [38]. Furthermore, in patients with HF whose self-care topics were taught by the teach-back method, the outcomes revealed that this self-care educational strategy may improve HF patients’ knowledge and performance, reduce readmission frequency, and increase the quality of life [39].

Targeted motivational interviewing during the nursing care of 93 patients with chronic HF in a randomized double-blind method resulted in increased scores of self-care maintenance and self-care management, as well as improved hospital readmission and mortality rate [40]. In addition, a randomized controlled trial elevating the effectiveness of the nurse-led home-based heart failure self-management program in patients with chronic HF suggested the program as a feasible and potentially effective strategy in improving chronic HF patients’ self-care management, behavior outcomes, and health-related quality of life [41].

Moreover, a randomized controlled trial showed the efficacy of an empowerment program on self-care behaviors and readmission of patients with HF [42].

Interestingly, a systematic review and meta-analysis study by Cañon-Montañez et al. [43] found that educational interventions could significantly reduce hospital readmission rates by 30% to 33%, as well as shorten the length of hospital stay by two days. Although adherence to HF self-care behaviors is effective in alleviating disease symptoms, increasing levels of quality of life, and reducing the rates of rehospitalization and mortality, it is documented that a large number of patients fail to implement ongoing self-care strategies in their daily lives [44]. Therefore, following up with patients, reviewing the provided education, and evaluating patients’ adherence to self-care behaviors can be accompanied by desired outcomes.

In this regard, several studies aimed to evaluate the effectiveness of predischarge educational interventions and postdischarge follow-up programs on hospital readmission and mortality rates in patients with HF. For example, Chen et al. [45] evaluated self-care behaviors, readmission, sleep quality, and depression in a longitudinal, nonequivalent two-group pretest-posttest study consisting of the intervention group (N=25), who received predischarge-tailored education and one-year follow-ups, and the control group (N=22), whose HF patients received routine predischarge HF education from direct care nurses only. The findings of the study revealed that in the intervention group, post-discharge self-care maintenance at 6 months and self-care management at 12 months were significantly improved. In addition, first and subsequent rehospitalization for the intervention group was fewer, but not significantly fewer, than the control group [45].

Also, an extended follow-up from a multisite randomized controlled trial consisting of intervention (HF patients and their family caregivers received HF self-care resources and educational intervention on self-care and management of HF symptoms during their index admission) and control groups (whose patients received routine education) revealed that intervention group had significantly lower rates of rehospitalization and mortality at 6 and 12 months [46]. These findings may directly suggest the desirable function of tailored educational interventions accompanied by follow-up programs in reducing the rate of re-hospitalization and death of patients with HF, and also indirectly, it can be argued that these interventions decrease the duration of hospitalization and health service costs. Nevertheless, the improvement of follow-up programs to support self-monitoring in patients with HF has attracted the attention of researchers [47] and has provided suggestions for policymakers to focus on improving primary care provider attachment rates and systems supporting informational continuity [48].

## Conclusion

The findings of the current review study indicated that the appropriate strategy of educational interventions before the discharge along with the improved post-discharge follow-up program may reduce the length of hospitalization and the rate of hospital readmission and mortality, as well as increase the quality of life in patients with HF. However, the comparison of currently recommended strategies and the agreement on the optimal approach of the educational intervention/follow-up program necessarily need to be determined by further studies.

## Conflict of Interest

None.
